# Vaginal metastasis as the initial presentation of leiomyosarcoma: a case report

**DOI:** 10.1186/s12885-017-3484-1

**Published:** 2017-07-26

**Authors:** Cecilia Villalaín-González, Álvaro Tejerizo-García, Patricia Lopez-Garcia, Gregorio López-González, Ma. Reyes Oliver-Perez, Jesús S. Jiménez-López

**Affiliations:** 1 Service of Obstetrics and Gynecology, Hospital Universitario 12 de Octubre, Avenida de Córdoba s/n, E-, 28041 Madrid, Spain; 2 Pathology Oncology Service, Hospital Universitario 12 de Octubre, Madrid, Spain

**Keywords:** Uterine neoplasm, Vaginal neoplasm, Leiomyosarcoma, Leiomyoma, Metastasis, Histopathology, Immunohistochemistry, Case report, Adjuvant chemotherapy, Gynecology

## Abstract

**Background:**

Uterine leiomyosarcomas are very rare and highly aggressive tumors that have a high rate of recurrence and poor prognosis, even when early diagnosed. Due to their relative rarity, there is limited research on optimal management strategies.

**Case presentation:**

A 60-year-old woman with a history of an asymptomatic uterine leiomyoma presented in October 2015 with postmenopausal bleeding and a friable vaginal cyst that bled when palpated. A partial cystectomy was performed, and malignant-like cystic and solid components were identified. Histopathology diagnosed an unclassifiable malignant epithelioid tumor. Subsequent imaging studies identified a malignant uterine tumor, a metabolically active vaginal lesion, and two benign leiomyomas. An anterior pelvic exenteration (colpectomy, hysterectomy, bilateral adnexectomy, total cystectomy, and cutaneous ureteroileostomy ad modum Bricker) were performed by laparotomy in March 2016. Examination of the surgical specimens identified a 75 × 75-mm leiomyoma, an 80 × 30-mm infiltrating mesenchymal uterine lesion with vascular invasion and tumor emboli, and a 60 × 30-mm perivascular vaginal tumor. Immunohistochemistry indicated a phenotypic transition from a uterine leiomyosarcoma to a vaginal epithelioid lesion; marker expression changed from the uterine tumor actin+/desmin+/caldesmon+/CD10− phenotype, through the tumor emboli, to an actin−/desmin−/caldesmon−/CD10+ phenotype in the vaginal lesion. A high-grade uterine mesenchymal tumor and vaginal metastasis were diagnosed. Adjuvant chemotherapy with docetaxel, gemcitabine, and doxorubicin commenced in May 2016 and treatment has been well tolerated.

**Conclusions:**

Differentiating leiomyosarcoma from leiomyoma is challenging and few tools other than microscopic evaluation are available. Vaginal compromise in leiomyosarcoma usually results from tumor extension, not hematogenous metastasis. A vaginal metastasis is a very rare initial presentation. We have found only two cases like this described on published literature. The atypical clinical and histological presentation in our case complicated diagnosis and delayed treatment. An early diagnosis and complete surgical clearance gives the best chance of survival, and imaging tools should be applied early in instances of new suspicious malignant lesions.

## Background

Uterine sarcomas are rare malignant tumors that account for just 1% of gynecological cancers, and for 3%–7% of malignant diseases of the uterine corpus. Their rarity and histopathological diversity make it difficult to determine their real prevalence although it is estimated to be about 3 to 7 in 100,000. The median age at diagnosis varies depending on the histological subtype, but it most frequently is between 40 and 60 years with mean age at diagnosis of 55 years [1, 2].

According to the histological classification, mesenchymal malignant uterine tumors can be categorized into seven subtypes [3, 4]. However, for practical terms, they are commonly divided into three groups: leiomyosarcomas, undifferentiated sarcomas, and endometrial stromal sarcomas. The prognoses of these histological subtypes vary, and leiomyosarcomas have one of the worst ones [3, 4].

Uterine leiomyosarcomas are aggressive tumors and have a high rate of local recurrence, as high as 70%, even in early disease stages. The histopathological characteristics of uterine leiomyosarcomas enable early invasion and widespread metastases, particularly to the lungs, liver, abdomen, pelvis and pelvic and para-aortic lymph nodes [5, 6]. Vaginal affection is usually the result of local primary tumor extension, not hematogenous dissemination.

The most distinctive histopathological characteristics of leiomyosarcomas are presence of coagulative tumour cell necrosis, cytological atypia, high mitotic rate and positive staining for muscle markers (actine, desmin, caldesmon). Leiomyosarcomas should be differentiated from mitotically active or atypical leiomyomas and uterine smooth-muscle neoplasms with low malignant potential. Coagulative tumour-cell necrosis is decisive and should be distinguished from hyaline and ulcerative necrosis. Main differences between uterine sarcomas can be found on Table 1 [7–10].

**Table 1:** Histological and immunohistochemical differences of uterine body tumors

	Uterine leiomyosarco	Endometrial stromal sarcoma	Smooth muscle tumors of uncertain malignant potential	Leiomyoma
Mitotic rate	>10MF/10HPF	Variable	<10MF/10HPF	<5MF/10HPF
Cell necrosis	Coagulative	Ischemic	Ischemic	Absent or Ischemic
Citological atypia	Frequent	Present	Present	Citologically bland
Vimentin	+	+	+	+
Actin	+	−	+	+
Desmin	++	−	+	+
Caldesmon	++	−	+	+
CD10	−	++	−	−

The following scale was used for staining reaction: - no staining

*MF* mitotic figures, *HPF* High power field

+ 1% to 25%, ++ 26% to 50%, and +++ 50% of the tumor cells stain positive

There is a consensus that uterine sarcoma treatment should be managed by an expert committee. Standard treatment for early stage uterine leiomyosarcomas is a simple hysterectomy with oophorectomy, without lymphadenectomy [6, 11]; the benefits of adjuvant chemotherapy remain controversial.

Women with intra abdominal involvement or distant metastases have a high risk of disease progression following surgery alone [11]. As such, adjuvant chemotherapy with doxorubicin or docetaxel and gemcitabine, combined with radiotherapy, has been suggested for tumors classified as stage II–III according to the International Federation of Gynecology and Obstetrics [11, 12]. Although the survival benefits of adjuvant chemotherapy are unclear, chemotherapy is frequently offered. For women with metastatic disease, who are not surgical candidates, palliative treatment is given; quality of life needs to be considered at every stage [6].

In this article, we report the case of a woman that presented to the emergency department with a vaginal friable cyst. Subsequent investigations indicated that this was a single uterine leiomyosarcoma metastasis. The atypical presentation in this case complicated and delayed diagnosis and treatment initiation. We have found only two cases like this described on published literature [13, 14]. On one of them the case was diagnosed after a vaginal biopsy on a 37-year-old patient that consulted because of abnormal uterine bleeding and abdominal distension. The other case was described on a 62-year-old patient that consulted because of postmenopausal bleeding and had a 2 cm polypoid mass on the lateral wall of the vagina that proved to be a leiomyosarcoma metastasis after biopsy. On both cases the histological characteristics of the metastasis were described as similar to the ones of the primary tumour and to the ones associated to uterine leiomyosarcomas.

## Case presentation

We present the case of a 60-year-old woman with a history of dyslipidemia, irritable bowel syndrome, and an asymptomatic uterine leiomyoma managed with clinical surveillance by general gynecology consultations. She had no prior history of surgery, and, apart from an aunt with postmenopausal breast cancer, had no family history of gynecological cancer. Her menarche was at 13 years and her menopause was at 50 years.

The patient’s annual follow-up gynecology consultations for leiomyoma growth control had been normal until October 2015. At this time, she presented to the emergency department with postmenopausal bleeding and at physical examination a small friable vaginal cyst on the anterior vaginal wall that bled when touched was identified. The cyst appeared to be the cause of the patient’s postmenopausal bleeding, although postmenopausal uterine bleeding could not be completely excluded. A gynecological physical exam was not possible because the bleeding, size and location of the cyst. Following initial examination, the patient was referred to Cervical Pathology Consults for further evaluation.

A pelvic examination performed as part of the cervical pathology consultation confirmed the presence of a 1.5 cm diameter cyst on the medial line of the anterior vaginal wall, 2–3 cm from the urethral meatus. Palpation of the cyst indicated that it was a rough, mobile, spherical mass with a distinct hardness and a friable surface that bled when touched. Surgical excision was recommended. In December 2015, a partial cystectomy was performed. Given the proximity of the cyst to the urethra, it was not possible to fully dissect its capsule and a complete cyst excision could not be achieved. During the intervention, the cyst ruptured and released cystic and solid components with malignant-like appearances.

Histopathological examination indicated an epithelioid neoplastic proliferation, with anaplastic forms, nuclear pleomorphism, and a high mitotic rate, that had infiltrated the large vessels. Immunohistochemistry showed positive staining for vimentin and CD10, but no cytokeratin (AE1-AE3, K903), hormone receptor (estrogen and progesterone), melanocyte marker (S200, Melan-A), vascular marker (CD31, CD34), Fli- 1, CD45, PGM-1, or alpha-inhibin expression. The histopathological diagnosis was an unclassifiable malignant epithelioid tumor; carcinoma and melanoma were excluded by the immunochemical analysis.

**Fig. 1:**
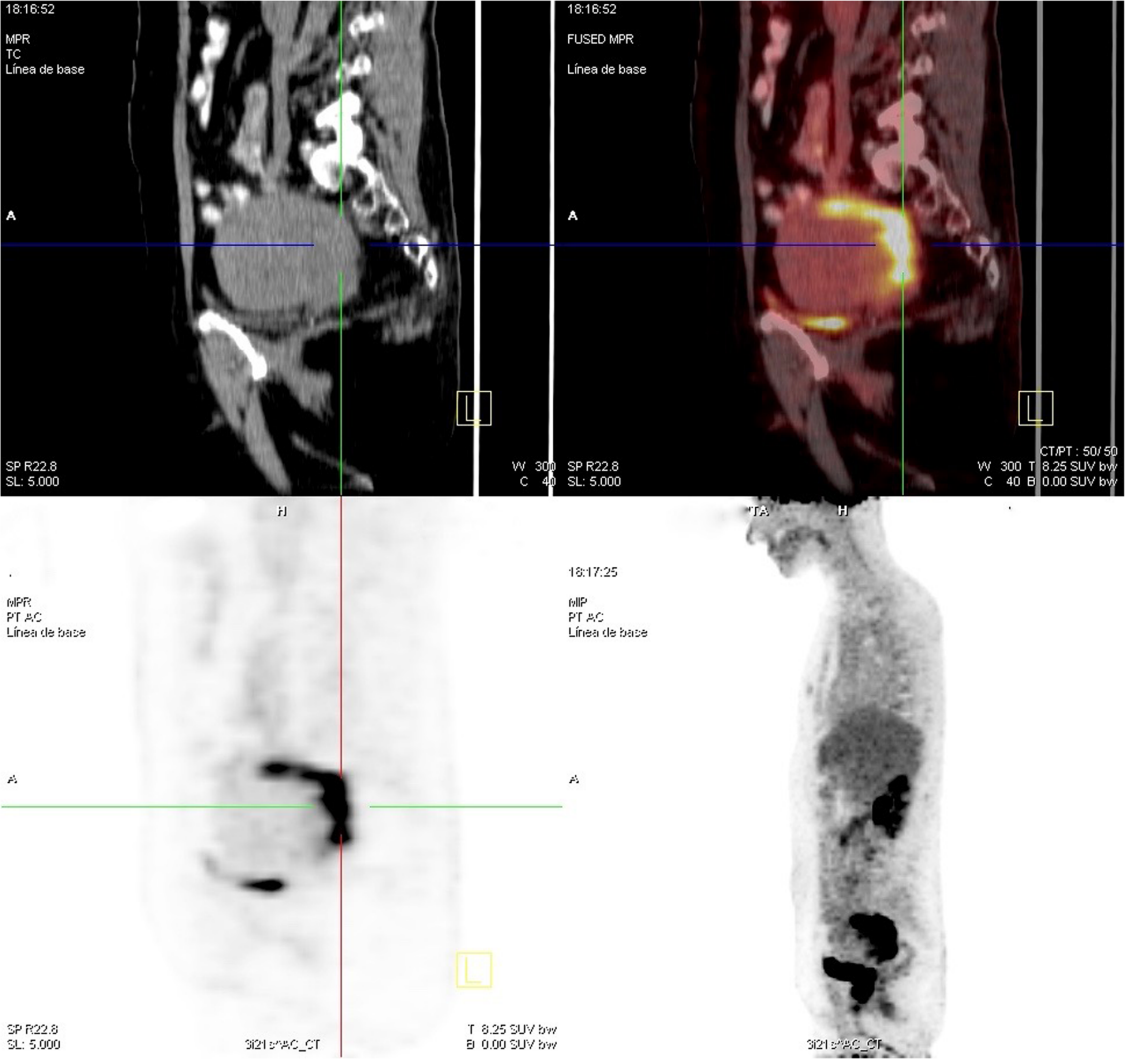
FDG-PET/CT demonstrates increased FDG uptake by the tumoral lesions on uterine corpus and vagina

A cystoscopy and positron emission tomography-computed tomography (PET-CT) were performed to determine whether the urethra was affected. The cystoscopic findings were normal. PET-CT indicated a malignant tumor that affected the uterine lower posterior-lateral left wall and Douglas pouch. A second metabolically active pathological lesion, which was suggestive of malignancy, was identified on the posterior wall and upper uterine segment. There were no signs of lymphatic locoregional or distant disease (Fig. 1). A 3D ultrasound and a hysteroscopic evaluation were planned to exclude any concomitant uterine pathology.

A transvaginal ultrasound demonstrated a 50-mm heterogeneous hyperechoic mass with undefined margins, which was similar to that observed with cervical neoplasms. The mass extended from the posterior cervical lip to the uterus. The presentation was atypical in that the mass was elongated in appearance. Two benign leiomyomas of 50-mm and 30-mm were also identified on the anterior uterine wall.

**Fig. 2:**
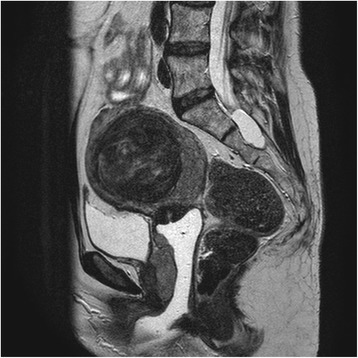
MRI showing a leiomyoma and posteriorly a mass that extends to the uterine isthmus-cervix juncture

A magnetic resonance imaging scan was subsequently performed. This showed the previously identified leiomyomas and a posterior right lateral 50 × 35.5 × 20-mm mass that overlapped the myometrium. The mass extended to the uterine isthmus cervical junction. No anomalies of the cervix or bladder wall and no locoregional disease, implants, ascites, or pelvic or inguinal adenopathies were identified (Fig. 2).

The hysteroscopic evaluation was not completed at the request of the patient, but a vaginoscopy enabled a further biopsy from the previous surgical site. The site continued to feel hard when palpated. Microscopic examination of the biopsy indicated a malignant proliferation that, similar to the previously removed vaginal cyst, only expressed vimentin and CD10 upon immunohistochemical analysis. These findings were inconclusive with regard to a histopathological diagnosis, but taken together with the radiological indication of a uterine body neoplasm, suggested that the patient had a secondary metastasis of a primary uterine tumor. Given the CD10+ immunohistochemistry results, this was thought to be an endometrial stromal sarcoma.

Although tumor marker analysis was not performed at this point, other laboratory tests indicated that the patient’s results were within reference range.

The case was presented at the Oncological Committee Meeting and a radical surgery approach, consisting of an anterior pelvic extenteration (colpectomy, hysterectomy, bilateral adnexectomy, total cystectomy, and cutaneous ureteroileostomy ad modum Bricker), was proposed.

Surgery was performed by laparotomy in March 2016. The immediate postoperative recovery was uneventful apart from anemia of 7.8 g/dL and oral candidiasis. 2 units of crossmatched compatible packed red blood cells and intravenous iron were transfused and an antifungal oral solution was prescribed. Surgical recovery was satisfactory and the patient was discharged on post-operative day 14 after being evaluated by the General Surgery, Urology, and Gynecology departments. Gross examination of the surgical specimen showed one 75 × 75-mm leiomyoma and two tumor lesions, one on the uterus and one at the vaginal level, with a leiomyosarcoma-compatible immunophenotype. The first tumor, which was 80 × 30 mm, was located on the posterior uterine aspect and extended from the uterine fundus to the lower segment, but it did not reach the cervix. It had completely infiltrated the myometrium, but not the uterine serosa (Fig. 1). It was a friable infiltrating tumor that was tan/white upon sectioning, had many areas of necrosis, and showed signs of peripheral vascular invasion. Signs of vascular invasion, with tumor emboli, were identified on the left parametrium, anterior vaginal wall, and right adnexa. The second vaginal tumor was a 60 × 30-mm polypoid lesion with an ulcerated surface. It was located on the anterior vaginal wall, close to the urethral vaginal septum, but it did not reach the urothelial mucosa.

**Fig. 3:**
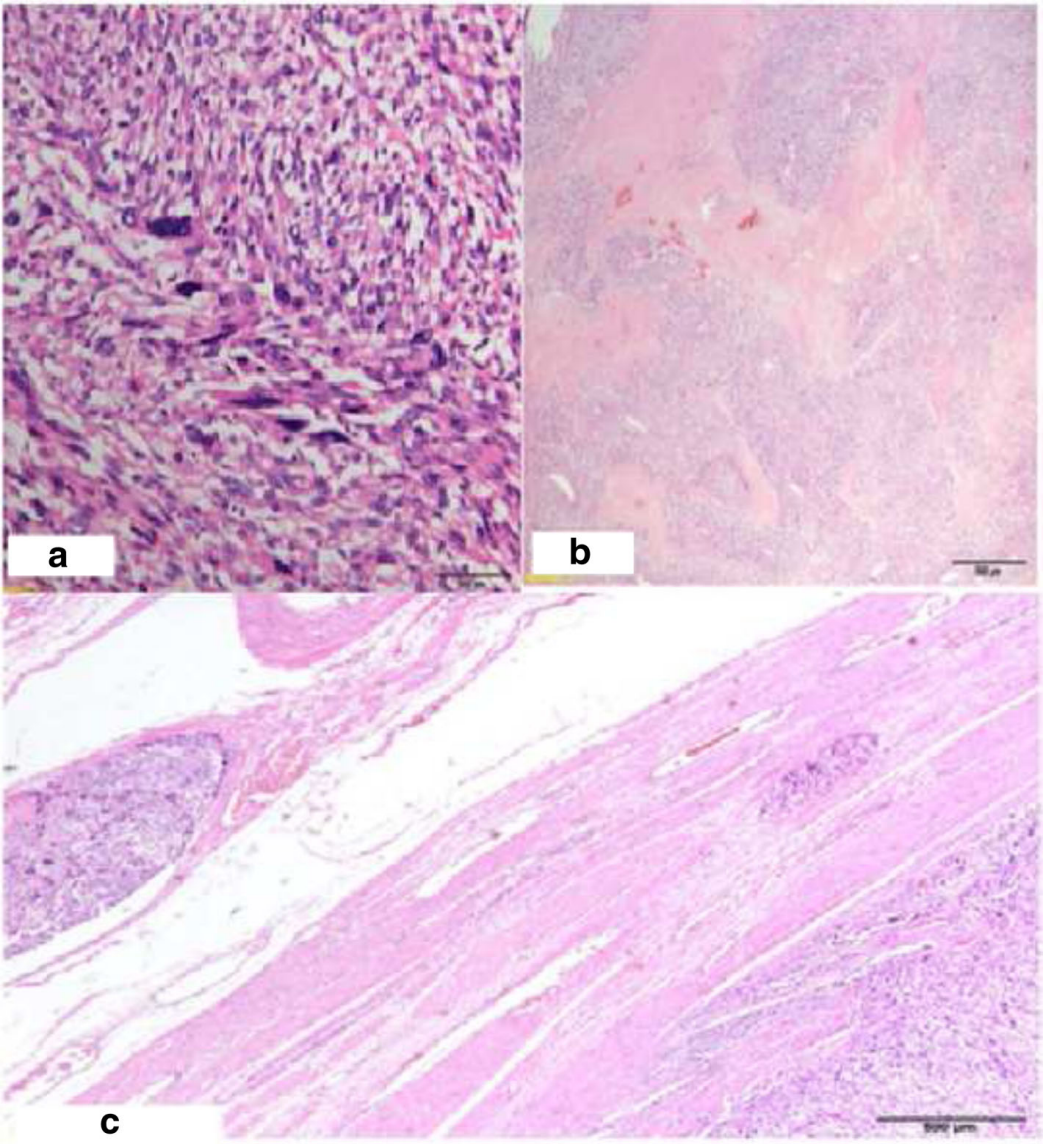
**a** Mesenchimal neoplasic proliferation with nuclear atypia, pleomorphism, prominent eosinophilic nucleoli and enlarged cytoplasm (× 200 hematoxylin/eosin). **b** Coagulative tumoral necrosis (×20 hematoxylin/eosin) (**c**) Tumoral emboli on myometrial vessels (×20 hematoxylin/eosin)

Histological section analysis indicated that the uterine lesion showed mesenchymal proliferation with large thick-walled muscular vessels and cleft-like spaces. It was a cellular tumor with spindle cells, nuclear atypia, prominent irregular nucleoli, moderate anisonucleosis, an enlarged cytoplasm, and eosinophilia (Fig. 3a). A high mitotic rate (28 mitoses/10 high power fields [HPF]) was observed (Fig. 3b), and the adjacent myometrial vessels showed many tumor emboli (Fig. 3c).

**Fig. 4:**
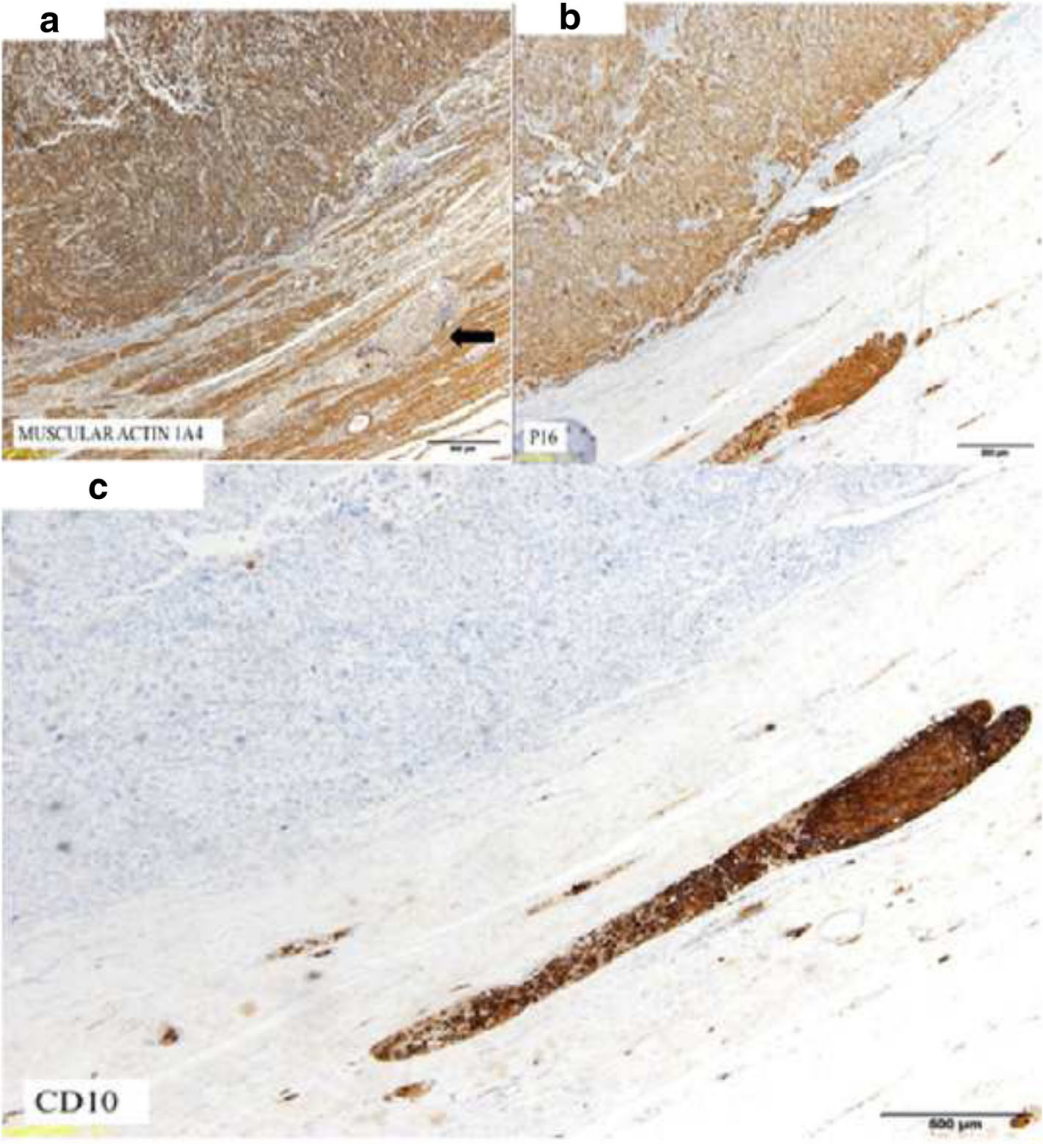
**a** Intense positive staining of endometrial tumoral cells for actin 1A4. Tumoral emboli are negative (marker). **b** Intense positive staining on tumoral cells on endometrial cells and tumoral emboli for p16 (**c**) Intense positive staining on tumoral cells on vascular tumoral emboli for CD10

Immunochemical analysis of the uterine lesion showed intense staining for actin A1-A4, actin HHF35, and caldesmon (Fig. 4a). Intense nuclear and cytoplasmic p16 staining was noted (Fig. 4b), and p53 overexpression was observed in the vascular tumor emboli and in 70% of the peripheral tumor cells of the main lesion. An analysis of CD10 expression indicated heterogeneous moderate staining in the main tumor lesion and homogeneous intense staining in the tumor emboli (Fig. 4c) The tumor cells were negative for all of the other markers investigated (pan-keratin AE1-AE3, hormone receptors, alpha-inhibin, calretinin, Melan A, WT1). The proliferative index (MIB-1) was 70%.

The uterine histological findings, indicating nuclear atypia, coagulative tumor necrosis, and a high mitotic rate, and the immunophenotype results were consistent with a diagnosis of leiomyosarcoma.

**Fig. 5:**
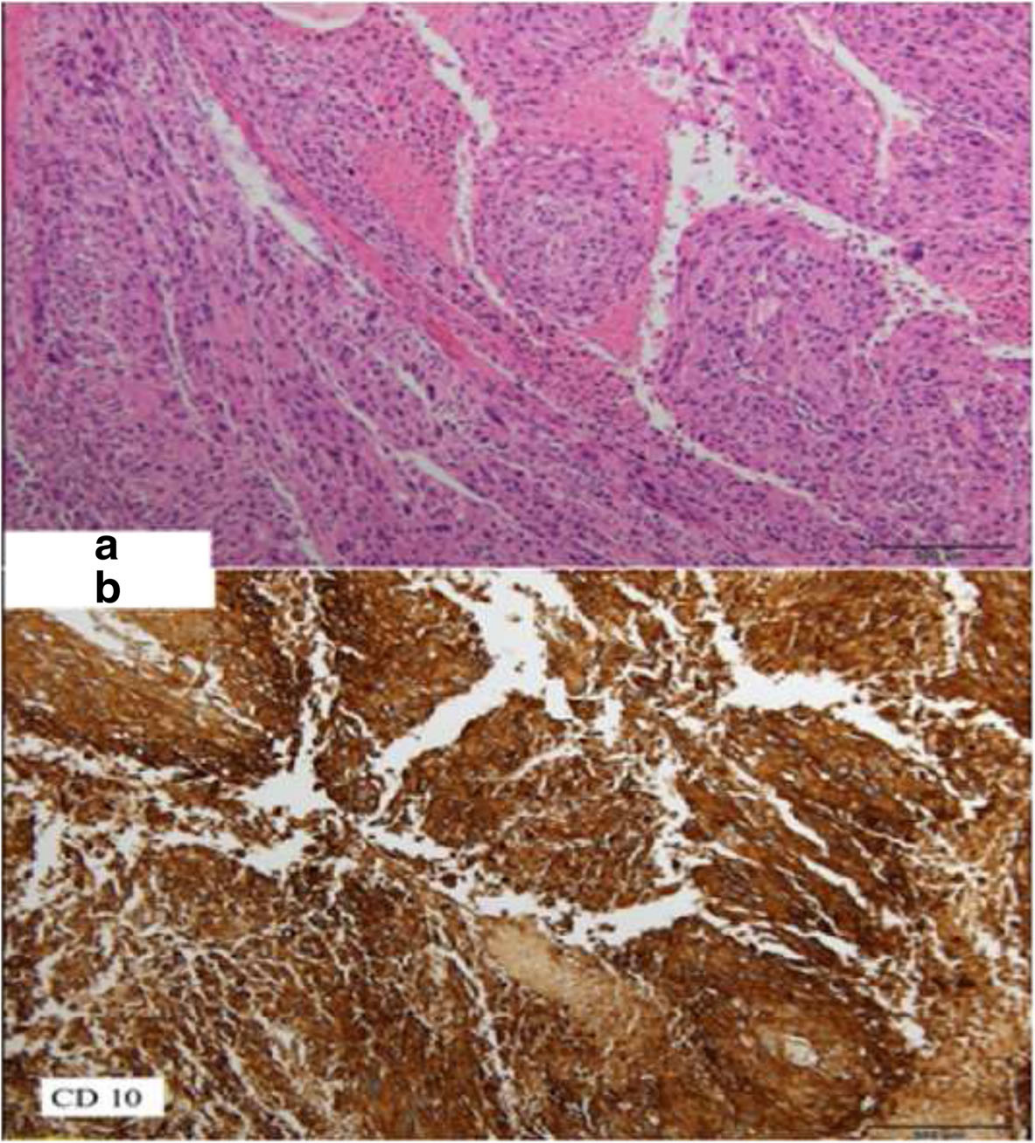
**a** Neoplasic proliferation constitued by epitheloid like cells. Tumoral necrosis. (×100 hematoxylin/eosin) (**b**) Intense positive staining on tumoral cells for CD10

Histological analysis of the vaginal lesion showed a neoplastic formation with nuclear atypia, pleomorphism, and prominent nucleoli. A high mitotic rate (17 mitoses/10 HPF) and coagulative tumor necrosis were also observed (Fig. 5a). CD34 and CD31 staining indicated that the neoplasm was perivascular with intravascular growth, and CD45 staining indicated a dense inflammatory lymphoid component. Immunochemical analysis of the lesion indicated intense cytoplasmic CD10 and p16 staining (Fig. 5b), and low desmin and Melan-A staining. Staining was also negative for other markers, including actin, caldesmon, and collagen.

Considering all of the findings, we diagnosed a uterine mesenchymal high grade tumor and a vaginal metastasis with an leiomyosarcoma-compatible immunophenotype.

The case was presented at the Oncological Committee Meeting again and radiotherapy was rejected because of its low effectiveness in these cases. The patient was referred to a medical oncologist to commence adjuvant chemotherapy with docetaxel, gemcitabine, and doxorubicin, which was started in May 2016. In May 2016, the tumor markers, Ca125, Ca19.9, CEA, and Ca15.3, were all negative. A PET-CT scan performed on the same day showed no evidence of locoregional recurrence or distant metastasis.

At the point of writing, no toxic side effects of chemotherapy have been observed and treatment is being well tolerated. Follow-up oncological gynecology consultations have been satisfactory with no signs of relapse, disease free for 12 months. The patient showed a good surgical recovery and had only minor complaints regarding vaginal dryness, which were treated with a topical moisturizer.

## Discussion and conclusions

Classically, leiomyosarcomas have been thought to be most common, and worthy of suspicion, in cases of rapidly growing leiomyomas or in patients who had previously received pelvic radiotherapy. However, recent studies have not upheld these assumptions [1, 2, 5, 15].

In the present case, the neoplasm had an atypical clinical and histological presentation. Clinically, the tumor was asymptomatic, with no signs of uterine compromise, and was only detected as a vaginal metastasis. Endometrial sarcomas most frequently present as abnormal uterine bleeding (70%–85% of cases), abnormal vaginal leucorrhea (10% of cases), or abdominal distention (8%–17% of cases). Excluding abnormal bleeding, findings in cases of leiomyosarcoma and leiomyoma [5, 15–17]. Distinguishing a leiomyoma from a uterine sarcoma is a challenge on the diagnostic process for all women with a uterine mass. Unfortunately, apart from a microscopic examination, no techniques have been validated for this to date. In our patient, the abnormal bleeding did not appear to be caused by classical metrorrhagia, but was attributed to a highly friable vaginal cyst. Given the unlikely nature of these onset characteristics, leiomyosarcoma was not considered as a first line diagnosis. Until the excision of the cyst there was no suspicion of malignancy, therefore no imaging techniques were performed prior to the surgery except for a chest X-Ray needed for anaesthetic preoperative evaluation.

Approximately 80% of vaginal cancers are secondary metastatic tumors. These usually derive from cervical (51%) or endometrial (13.3%) primary tumors [13, 14, 18]. Other urogenital tumors that have been shown to metastasize to the vagina are kidney tumors (1.3%), ovarian tumors, choriocarcinoma, and bladder cancer. In cases of leiomyosarcoma, vaginal compromise is usually caused by tumoral extension, and not hematogenous metastasis; as described before, we have only found two cases like ours described in the literature [13, 14].

Women with leiomyosarcoma have a poor prognosis regardless of stage. In a large study of women with leiomyosarcoma that included 1396 patients, stratified by stage, the five-year disease specific overall survival for stage I, II, III, and IV disease was 76, 60, 45, and 29%, respectively. In our case, given that we found a vaginal metastasis, we faced a stage IV leiomyosarcoma [19].

Most clinical guidelines [6, 7, 11] agree that uterine sarcomas should be managed with radical surgery (hysterectomy and bilateral salpingo-oophorectomy). For women with extrauterine disease, a surgical approach is preferable if a complete resection is feasible. There is little information on management of metastasis on primary surgery as most studies focus on clinical practice on recurrence. Because most uterine leiomyosarcomas portend and aggressive growth, resection of metastasis will benefit only a highly selected group such as those with solitary metastasis [20–23], as it was our case. Adyuvant treatment should be decided by an expert oncological committee.

In our case, the tumour was confined to the uterus and there was a single vaginal metastasis, making complete resection feasible. Although it is not the stablished procedure, we decided to perform an anterior pelvic exenteration due to the proximity of the tumor to the bladder (even though on the PET-CT and on the cystoscopy tumoral infiltration was not observed, microinfiltration could not be completely ruled out). The surgical specimen included both the primary tumour and the metastases**.**

Adjuvant chemotherapy with docetaxel, gemcitabine and doxorubicin was decided by the expert panel at our Oncological Committee Meeting. This treatment is in accordance with the most recently published research [24, 25]. Radiotherapy was rejected because of its low performance in metastatic disease cases [26–28].

Mesenchymal proliferations of the uterine body can be categorized into two groups: those with smooth muscle differentiation (including leiomyomas, smooth muscle neoplasms of uncertain malignant potential, and leiomyosarcomas), and those with endometrial stromal differentiation (including endometrial stromal nodules, low grade endometrial stromal sarcomas, and high grade endometrial stromal sarcomas). The presence of coagulative tumor cell necrosis, cytological atypia, a high mitotic rate, and positive staining for muscle markers (desmin, and caldesmon) are diagnostic criteria for leiomyosarcoma. Intense staining for CD10 is usually associated with endometrial stromal sarcomas [8–11] and it progressively acquired different neoplastic features. Our histological analysis indicated that nuclear pleomorphism and cellular atypia in the uterine lesion became more frequent until epithelioid characteristics, similar to the vaginal lesion, were achieved.

Immunochemical marker expression changed from the uterine immunophenotype (actin+, desmin+, caldesmon+, CD10+), through the tumor emboli, to the vaginal immunophenotype (actin−, desmin−, caldesmon−, CD10+). Through the immunohistochemical analysis, one can observe the transition from the uterine to the vaginal lesion; acquiring a different immunophenotype, but remaining part of the same tumor [29, 30]. From these findings, we have assumed that the vaginal neoplasm represents hematogenous spread of the uterine body neoplasm; although the vaginal neoplasm has a different immunophenotype, the transition from the uterine to the vaginal tumor can be observed in the vascular tumor emboli. It is possible that these different morphologies and immunochemistry staining patterns are due to non-coding RNAs, which post-transcriptionally regulate gene expression, and have been observed in leiomyosarcomas and endometrial stromal sarcomas [31–34]. On Table 2 we show the main differences between leiomyosarcomas, endometrial stromal sarcomas, our case uterine leiomyosarcoma and its vaginal metastasis.

**Table 2:** Differences between leiomyosarcoma, endometrial stromal sarcoma, our case of uterine leiomyosarcoma and vaginal metastasis

	Uterine leiomyosarcoma	Endometrial stromal sarcoma	Case: Uterine leiomyosarcoma	Case: Vaginal metastasis
Mitotic rate	>10MF/10HPF	Variable	28MF/HPF	17MF/HPF
Coagulative cell necrosis	Coagulative	Ischemic	Coagulative	Coagulative
Citological atypia	Frequent	Present	Present	Present
Vimentin	+	+	+	+
Actin	+	−	+++	−
Desmin	++	−	+++	−
Caldesmon	++	−	+++	−
CD10	−	++	−	++

The following scale was used for staining reaction: - no staining

*MF* mitotic figures, *HPF* High power field

+ 1% to 25%, ++ 26% to 50%, and +++ 50% of the tumor cells stain positive

Non coding RNAs are being linked more frequently to uterine sarcomas and practitioners should bear them in mind when facing an atypical histopathology.

The atypical immunohistochemical analysis of the metastasis entangled a diagnosis at first, delaying treatment.

Leiomyosarcomas are very rare tumors. They are typically diagnosed in peri- and post-menopausal women aged 51–56 years, but they can occur in adults of any age. The progression of leiomyosarcomas is rapid and aggressive, and they are associated with a high rate of local recurrence even when they are identified at an early stage. An early diagnosis and complete surgical clearance gives the best chance of survival; achieving this more frequently depends on further development of imaging techniques and the discovery of biochemical and molecular markers that can increase the preoperative detection of early stage disease.

We believe that this case is relevant because of its extremely rare presentation and its unique histopathological staining. As previously stated, this staining is most probably associated to non-coding RNAs post-transcriptionally regulating gene expression in leiomyosarcomas and endometrial stromal sarcomas.

Imaging for diagnosis and staging, and a second biopsy were key to reaching an initial diagnostic approach in our case. The final pathology report indicated an hematogenous spread of a uterine leiomyosarcoma, which had changed its immunophenotype through metastasis.

When faced with new suspicious malignant vaginal lesions, diagnostic techniques should not be delayed. Although unlikely, metastasis of an aggressive, fast growing tumor with a poor prognosis, like a leiomyosarcoma, is possible.

## Abbreviations

HPF: High power fields
PET-CT: Positron emission tomography-computed tomography
